# Effects of a home-exercise therapy programme on cervical and lumbar range of motion among nurses with neck and lower back pain: a quasi-experimental study

**DOI:** 10.1186/s13102-015-0025-6

**Published:** 2015-12-04

**Authors:** Tiina Freimann, Eda Merisalu, Mati Pääsuke

**Affiliations:** Tartu University Hospital, Puusepa 8, Tartu, 51014 Estonia; Department of Public Health, Medical Faculty, University of Tartu, Ravila 14a, Tartu, 50411 Estonia; Institute of Technology, Estonian University of Life Sciences, Kreuzwaldi 56, Tartu, 51014 Estonia; Institute of Exercise Biology and Physiotherapy, University of Tartu, Ravila 14a, Tartu, 50411 Estonia

**Keywords:** Cervical range of motion, Lumbar range of motion, Musculoskeletal pain, Exercise therapy

## Abstract

**Background:**

Cervical and lumbar range of motion limitations are usually associated with musculoskeletal pain in the neck and lower back, and are a major health problem among nurses. Physical exercise has been evaluated as an effective intervention method for improving cervical and lumbar range of motion, and for preventing and reducing musculoskeletal pain. The purpose of this study was to investigate the effects of a home-exercise therapy programme on cervical and lumbar range of motion among intensive care unit nurses who had experienced mild to moderate musculoskeletal pain in the neck and or lower back during the previous six months.

**Methods:**

A quasi-experimental study was conducted among intensive care unit nurses at Tartu University Hospital (Estonia) between May and July 2011. Thirteen nurses who had suffered musculoskeletal pain episodes in the neck and or lower back during the previous six months underwent an 8-week home-exercise therapy programme. Eleven nurses without musculoskeletal pain formed a control group.

Questions from the Nordic Musculoskeletal Questionnaire and the 11-point Visual Analogue Scale were used to select potential participants for the experimental group via an assessment of the prevalence and intensity of musculoskeletal pain. Cervical range of motion and lumbar range of motion in flexion, extension, lateral flexion and (cervical range of motion only) rotation were measured with a digital goniometer. A paired *t*-test was used to compare the measured parameters before and after the home-exercise therapy programme. A Student’s *t*-test was used to analyse any differences between the experimental and control groups.

**Results:**

After the home-exercise therapy, there was a significant increase (*p* < 0.05) in cervical range of motion in flexion, extension, lateral flexion and rotation, and in lumbar range of motion in lateral flexion. Cervical range of motion in flexion was significantly higher (*p* < 0.01) in the experimental group compared to the control group after therapy.

**Conclusions:**

Our results suggest an 8-week intensive home-exercise therapy programme may improve cervical and lumbar range of motion among intensive care nurses. Further studies are needed to develop this simple but effective home-exercise therapy programme to help motivate nurses to perform such exercises regularly.

**Trial registration:**

Current Controlled Trials ISRCTN19278735. Registered 27 November 2015.

## Background

Cervical range of motion (CROM) and lumbar range of motion (LROM) limitations are usually associated with musculoskeletal pain (MSP) in the neck and lower back, and is a major health problem among intensive care unit (ICU) nurses [1, 2]. Earlier studies have indicated a high frequency of neck and lower back pain among Estonian nurses [3], necessitating a search for appropriate intervention methods to improve CROM and LROM in order to prevent and reduce neck and lower back pain among nurses.

Physical exercise has been evaluated as an effective intervention method for improving CROM and LROM [4], and for reducing MSP in the neck and lower back [5, 6]. Many authors have emphasized that such programmes should be conducted with sufficient frequency, intensity and duration, plus appropriate ergonomic counselling and supervision [7, 8]. Ergonomic interventions without physical exercise may be ineffective in preventing or reducing MSP [9, 10], possibly because of insufficient or inconsistent implementation of ergonomic practices [11]. Some studies have supported physical activity programmes in the workplace as being more effective intervention methods compared to home-exercise programmes [6]. However, Jakobsen and et al. [12] found the effects of physical exercise on musculoskeletal pain did not depend on whether exercises were conducted at the workplace or during leisure time at home. Moreover, Kuukkanen et al. [13] found that supervised home exercises led to reduced lower back pain and these positive effects persisted for more than five years after the exercise programme was compleated. Some studies have found positive results of physical exercise when the training period lasted longer than 10 weeks [8].

The literature review by Dawson et al. [14] provides conflicting evidence regarding the efficacy of physical exercise to prevent lower back pain among nurses. At present there is no simple and cost-effective physical exercise programme for improving cervical and lumbar ROM, and reducing neck and lower back pain, among nurses.

The aim of our study was to investigate the effects of an 8-week home-exercise therapy programme that included ergonomic counselling and supervision, on CROM and LROM among ICU nurses who had suffered episodes of mild to moderate MSP in the neck and or lower back during the previous six months. We hypothesized that our specially designed home-exercise therapy programme could improve cervical and lumbar range of motion to reduce neck and lower back pain among ICU nurses.

## Methods

### Subjects and settings

**Fig. 1:**
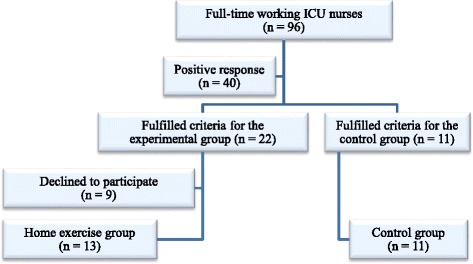
Participant flowchart

This case control series study was conducted among ICU nurses (*n* = 96) at Tartu University Hospital (Estonia) between May and July 2011. Twenty-two female intensive care nurses from three different ICUs fulfilled the eligibility criteria for the experimental group, and thirteen completed the 8-week home-exercise therapy programme. The other nine participants gave up due to health reasons, a change of residence or a lack of time. Eleven nurses fulfilled the eligibility criteria for the control group and registered to participate in the study. A participant flowchart is provided in Fig. 1. The study was approved by the Research Ethics Committee of the University of Tartu (protocol number 202 T-19) and conducted in accordance with the Helsinki Declaration. All subjects were informed about the purpose and content of the study and provided written consent.

Inclusion criteria for the experimental group were: having worked full time for at least a year in the ICU; being under 40 years of age; having a body mass index <32; having experienced mild to moderate pain in the cervical and or lumbar regions during the previous six months. Inclusion criteria for the control group were: being under 40 years of age; having a body mass index <32; experiencing no MSP during the previous six months. Exclusion criteria for both groups were acute orthopaedic and or neurological diseases and pregnancy.

### The exercise therapy programme

**Fig. 2:**
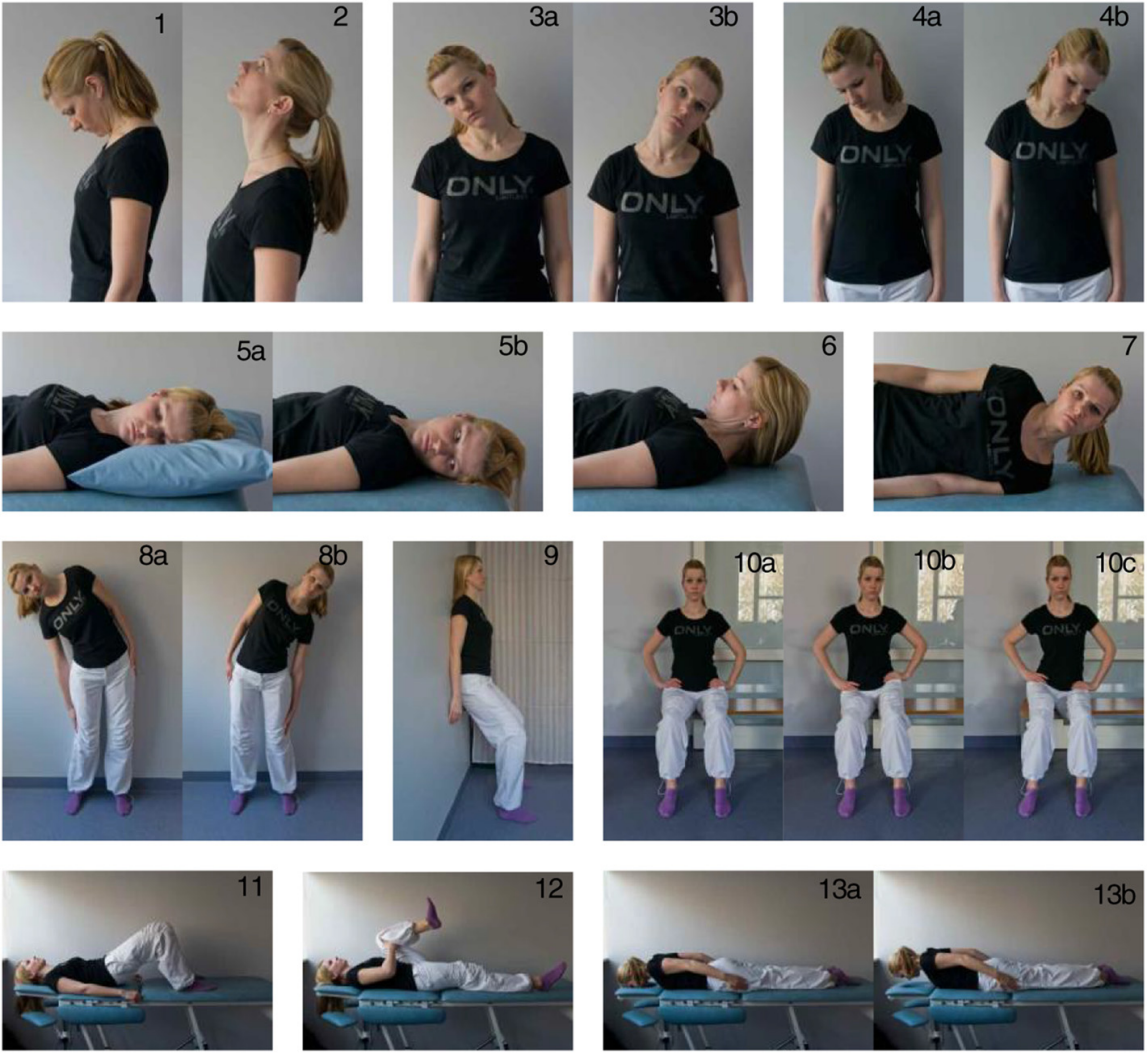
Examples of the stretching and strengthening exercises used in the home-exercise therapy programme. * The subject of this figure gave consent for these photos to be reproduced here

The home-exercise therapy programme was chosen for the experimental group because of its simplicity and low cost compared with workplace exercise programmes [15]. It was important that the participants would be able to perform the exercises by themselves, i.e. without continuous supervision by a physiotherapist. Based on previous studies [4, 16, 17], effective stretching and strengthening exercises for the cervical and lumbar region were included in the exercise therapy programme (Fig. 2).

The experimental group underwent 8-weeks of exercise therapy, with the frequency and intensity of the exercises increasing every two weeks. The training load progressively increased according to the principle of the gradual rising of loads [18]. The participants were asked to perform exercises as one to three sets of 8–10 repetitions. Exercises 4a, 4b and 12 (both feet) were performed with 2–3 repetitions per set. The goal for subjects was to perform exercises once a day, six days a week, for 8 weeks. Each session lasted from 20 min in the first two weeks to 60 min in the last two weeks. The control group were asked to continue their normal life.

Before the start of the home-exercise programme, the experimental group underwent training with the guidance of a physiotherapist regarding the specific ergonomic considerations and correct techniques for performing the exercises at home. Ergonomic guidance was also given for correct working postures and patient handling tasks. The experimental group received written materials that included photographs depicting the correct techniques of the exercise therapy. After four weeks the experimental group met with a programme supervisor (physiotherapist), who examined their technique and resolved any problems related to the programme.

### Data collection

A questionnaire was prepared for the selection of potential participants, which included questions from the Nordic Musculoskeletal Questionnaire (NMQ) [19] and the 11-point Visual Analogue Scale (VAS) [20]. The prevalence and intensity of MSP lasting for longer than a day in the neck and or lower back during the previous six months was assessed [20]. Mild to moderate MSP constituted the inclusion criteria for the experimental group. Throughout the programme the experimental group completed a training diary, which contained a detailed description of time spent on each session, the exercises conducted and a description of any other aerobic training not included in the study programme. The control group continued their normal life and did not keep an exercise diary.

### Measurements

CROM in flexion, extension, lateral flexion and rotation, and LROM in flexion, extension and lateral flexion, were measured using a digital goniometer (AcumarTM Digital Inclinometer, Version 5.0), which is an objective and reliable method [21–23] recommended by international clinical guidelines [24, 25]. All CROM and LROM measurements in the experimental group were taken before and after the 8-week exercise therapy programme; measurements for the control group were taken at the same time as the second measurements for the experimental group. All the measurements were made by the same physical therapist and researcher to achieve a high reliability of measurements [22]. The best of three performances was used for each flexion, extension and rotation measurement.

### Statistical analysis

Statistical analyses were carried out using the Statistical Package for the Social Sciences (SPSS) Version 18.0. First, descriptive statistics were used to analyse the data. The means and standard deviations of each measurement per group were calculated. A paired *t*-test was used to compare CROM and LROM before and after the home-exercise therapy programme; a Student’s *t*-test was used to examine any differences between the experimental and control groups. The significance level for all tests was set at *p* < 0.05.

## Results

The age ranges of the experimental and control groups were 23–37 (*n* = 13) and 22–39 (*n* = 11) years old respectively. Both groups were homogeneous (*p* > 0.05) for the anthropometric variables measured (Table 1).

**Table 1:** Comparison of age and anthropometric parameters in the experimental and control groups

Features	Experimental group (*n* = 13)		Control group (*n* = 11)		
	Mean	SD	Mean	SD	p
Age (yrs)	29.2	5.1	31.1	5.1	0.414
Height (cm)	165.6	4.8	167.5	3.6	0.191
Weight (kg)	66.5	2.4	67.8	3.0	0.885
BMI (kg/m^2^)	24.2	0.9	24.2	1.1	0.977

Eight of the nurses in the experimental group had experienced both neck and lower back pain, two of them only neck pain and three only lower back pain. The mean pain intensity score (using the 11-point VAS) over the six months was 4.1 (SD 2.5). According to their training diaries, the experimental group conducted home-exercise therapy on average five times per week over the 8-week period, with the duration of exercising ranging from 20 min per session in the first two weeks to 60 min per session in the last two weeks.

Table 2 presents the means and standard deviations for CROM and LROM for each group, *p*-values for the comparison of variables before and after the exercise therapy program and differences between the experimental and control groups. Statistically significant differences were found in the experimental group’s CROM values before and after therapy. There was a significant increase in cervical flexion (26 %; 14.7°; *p* = 0.000), extension (18 %; 10.7°; *p* = 0.002), right lateral flexion (15 %; 6.0°; *p* = 0.002), left lateral flexion (14 %; 5.9°; *p* = 0.012), right rotation (10 %; 8.5°; *p* = 0.002) and left rotation (9 %; 7.4°; *p* = 0.010). After therapy CROM in flexion was significantly higher in the experimental group compared to the control group (11 %; 6,5°; *p* = 0.004). Significant increases in lumbar right lateral flexion (20 %; 5.5°; *p* = 0.003) and left lateral flexion (17 %; 5.1°; *p* = 0.009) after therapy were also found.

**Table 2:** Cervical and lumbar range of motion values in the experimental and control groups and *p*-values for the comparison of differences within and between groups

	Experimental group			Control group		
	Mean^a^ (SD)	Mean^b^ (SD)	p	Mean (SD)	p^c^	p^d^
Cervical ROM						
Flexion	42.3 (8.1)	57.0 (7.0)	0.000	50.5 (7.6)	0.109	0.004
Extension	49.5 (10.7)	60.2 (9.2)	0.002	58.4 (13.5)	0.080	0.704
Lateral flexion (right)	34.8 (4.5)	40.8 (5.5)	0.002	37.6 (6.2)	0.133	0.203
Lateral flexion (left)	35.5 (6.6)	41.4 (7.8)	0.012	39.9 (7.1)	0.107	0.634
Rotation (right)	75.7 (8.7)	84.2 (6.4)	0.002	83.2 7.3)	0.323	0.731
Rotation (left)	78.4 (8.8)	85.8 (7.6)	0.010	84.8 (6.1)	0.354	0.766
Lumbar ROM						
Flexion	62.2 (9.8)	61.2 (7.2)	0.698	60,0 (7.9)	0.275	0.691
Extension	22.4 (7.9)	23.6 (5.9)	0.528	28.2 (7.6)	0.090	0.113
Lateral flexion (right)	21.9 (4.7)	27.4 (2.2)	0.003	27.2 (7.4)	0.045	0.925
Lateral flexion (left)	24.5 (6.0)	29.6 (3.6)	0.009	27.6 (5.4)	0.142	0.272

^a^experimental subjects before therapy; ^b^experimental subjects after therapy; ^c^experimental subjects before therapy compared to controls; ^d^experimental subjects after therapy compared to controls

*p* < 0.05 constituted significant differences within and between groups

## Discussion

This study evaluated the effects of an 8-week home-exercise therapy programme on CROM and LROM among ICU nurses. We found a significant increase in cervical flexion, extension, lateral flexion and rotation, and in lumbar lateral flexion, after therapy. The largest increase in range of motion was found for cervical flexion; after 8-weeks, this value approached the age-specific normal CROM value of 58–60° [26]. Our study results showed the exercise therapy programme had a greater effect on CROM compared to LROM. Therefore, the types of exercises needed to achieve better results in both CROM and LROM within a single therapy programme should be explored further. Our study results are difficult to compare with previous findings due to differences in study designs, exercise therapy programmes and outcome measurements. Only in some previous studies are CROM and or LROM compared before and after an exercise therapy programme. Tseng et al. [27] examined the effects of range-of-motion exercise on the upper and lower extremities and found positive effects in enhancing physical and psychological functions of older people after a stroke. More evidence is available concerning the positive effects of regular exercise on musculoskeletal symptoms [28–30]. Alexandre et al. [29] suggested that regular exercise can reduce musculoskeletal symptoms in nursing personnel. However, Dehlin et al. [31] found no effect of eight weeks of physical exercise on lower back pain. The negative results in their study could be explained by the low frequency of physical exercises, because in the study exercise was performed only twice a week and may thereby have been insufficient to decrease MSP. Cleland et al. [32] found that thoracic spine manipulation together with cervical range of motion exercise was more beneficial for patients with neck pain than exercise only. Since neck and lower back pain are often associated with CROM and LROM limitations, the results of previous studies allowed us to make assumptions about how physical exercise led to CROM and LROM improvements. After the exercise therapy programme in our study, six participants were completely pain-free; and on mean pain intensity in the neck and lower back of the other seven participants was significantly lower than before (0.7 (SD 0.7).

Our method of investigation had the advantage of using well-known instruments for collecting MSP self-evaluations (NMQ and VAS); however Numerical Pain Rating Scale (NPRS) have been found to be more clinimetrically reliable than VAS [33, 34]. We also used an objective and reliable method for collecting CROM and LROM measurements (AcumarTM Digital Inclinometer, Version 5.0) [21–23]. Additionally, as all CROM and LROM measurements were made by the same physical therapist, a high reliability of measurements can be assumed [22].

The main limitations of this study were related to the number of subjects and quasi-experimental design of the study. Due to the small population of volunteer ICU nurses, it was difficult to find a sufficient sample size of subjects with suitable characteristics for randomization. In general, the major problem with quasi-experimental designs is that the experimental and control group might not be similar in terms of the demographic parameters selected for intervention and comparison. This lack of recruitment numbers required the control group to be from an asymptomatic population. However, the experimental and control group in our study were homogeneous for age, height, weight and BMI (Table 1). Our study was limited to investigating the effects of a home-exercise therapy programme on CROM and LROM. Other intervention strategies (e.g. participatory ergonomics) that could increase the effectiveness of any programme should also be taken into consideration [35]. As the aetiology of cervical and lumbar disorders is multifactorial, a combination of intervention programmes for intensive care nurses should be studied in the future [36]. However, as the cost-effectiveness of any intervention method is an important consideration, it would be ideal to find effective and low-cost interventions [15].

According to the training diaries nine of the experimental subjects participated in other aerobic training, such as cycling, running, walking, swimming, rollerblading and rowing, on average two times a week. Aerobic training outside of the exercise therapy programme was negatively associated with total CROM. This association might show possible over-training and or physical overload in the cervical spine, which may have influenced the effectiveness of the programme. In the future, randomized controlled trials are recommended to investigate the effects of exercise therapy programmes on CROM and LROM with and without other types of aerobic training. Consideration of the duration of the exercise program, initiating at 20 min and increasing to 60 min, may also need to be considered in terms of participant demand.

Our study results confirm that home-exercise programmes are useful for improving cervical and lumbar range of motion. To achieve better results in mobility of lumbar flexion and extension parameters, specific exercises individually adapted for each person need to be implemented in further studies. Although Alexandre et al. [29] suggest that a standard programme of exercises conducted twice a week with an ergonomic approach could reduce musculoskeletal symptoms in nursing personnel, an individual approach could be more effective. Jakobsen et al. [12] found that performing physical exercise at the workplace together with colleagues may be more motivating for some employees.

## Conclusions

The present study indicated that CROM increased in all studied directions and that LROM increased in lateral flexion after the completion of an 8-week home-exercise therapy programme designed for intensive care nurses with MSP. We conclude that the used home-exercise therapy programme seemed to be effective for improving CROM and to a lesser extent LROM. Further studies are needed to develop this simple but effective home-exercise therapy programme to help motivate nurses to perform such exercises regularly. This study may contribute to the knowledge base within this area, however further research is required in larger samples, possibly with a symptomatic control group and more clinically rigorous outcome measures.

## Abbreviations

CROM: Cervical range of motion
LROM: Lumbar range of motion
MSP: Musculoskeletal pain
ICU: Intensive care unit
NMQ: Nordic Musculoskeletal Questionnaire
VAS: Visual Analogue Scale
